# Early development of *Drosophila* embryos requires Smc5/6 function during oogenesis

**DOI:** 10.1242/bio.019000

**Published:** 2016-06-10

**Authors:** Martin Tran, Vasilios Tsarouhas, Andreas Kegel

**Affiliations:** 1 Department of Cell and Molecular Biology, Karolinska Institutet, Stockholm S-17177, Sweden; 2 Department of Molecular Bioscience, The Wenner-Gren Institute, Stockholm University, Stockholm S-10691, Sweden

**Keywords:** Smc proteins, DNA DSBs, Chromosomes, Anaphase bridges, Pachytene arrest, Karyosome

## Abstract

Mutations in structural maintenance of chromosomes (Smc) proteins are frequently associated with chromosomal abnormalities commonly observed in developmental disorders. However, the role of Smc proteins in development still remains elusive. To investigate Smc5/6 function during early embryogenesis we examined *smc5* and *smc6* mutants of the fruit fly *Drosophila melanogaster* using a combination of reverse genetics and microscopy approaches. Smc5/6 exhibited a maternally contributed function in maintaining chromosome stability during early embryo development, which manifested as female subfertility in its absence. Loss of Smc5/6 caused an arrest and a considerable delay in embryo development accompanied by fragmented nuclei and increased anaphase-bridge formation, respectively. Surprisingly, early embryonic arrest was attributable to the absence of Smc5/6 during oogenesis, which resulted in insufficient repair of pre-meiotic and meiotic DNA double-strand breaks. Thus, our findings contribute to the understanding of Smc proteins in higher eukaryotic development by highlighting a maternal function in chromosome maintenance and a link between oogenesis and early embryogenesis.

## Abstract (precis)

**Summary:** Early emerging problems during oogenesis, such as DNA double-strand breaks, can affect chromosome duplication and segregation in embryogenesis in *Drosophila*. Moreover, environmental cues including temperature are important for proper oogenesis.

## INTRODUCTION

During the development of multicellular organisms such as insects, worms, and mammals, chromosomes are coordinated for replication, transcription, repair, and segregation. Therefore, conserved proteins critical to maintaining chromosome dynamics, organization, and integrity throughout these processes have evolved.

Structural maintenance of chromosomes (Smc) proteins belong to an evolutionarily conserved protein family that assemble into three major and distinct complexes. In eukaryotes, the sister-chromatid cohesion (SMC1/3)-, the chromosome condensation (SMC2/4)-, and the SMC5/6-complex regulate nearly all aspects of chromosome biology to ensure genome integrity (Hirano, 2002). The Smc5/6 complex, hereby referred to as Smc5/6, is the least characterized complex of all three. Smc5/6 is implicated in DNA recombination and repair, chromosome replication and segregation, and resolution of joint molecules and other replication-induced topologically complex DNA intermediates (De Piccoli et al., 2009; Kegel and Sjogren, 2010; Murray and Carr, 2008; Wu and Yu, 2012). In yeast, Smc5/6 consists of Smc5, Smc6, and six non-SMC elements (Nse1-6) (Duan et al., 2009), whereas in *Drosophila* these counterparts are less described. Recent studies on *Drosophila* suggested a conserved role of Smc5/6 in genome maintenance. Heterochromatic DNA double-strand break (DSB) repair required Smc5/6 function to prevent aberrant recombination in cultured S2 cells, whereby loss-of-function caused caffeine-induced apoptosis of developing imaginal discs (Chiolo et al., 2011; Li et al., 2013); however the function of Smc5/6 during early *Drosophila* development remains unknown. Since *SMC6* knockouts in mice are embryonic lethal (Ju et al., 2013) further investigations are required to reveal the importance of Smc5/6 in early development.

Successful embryogenesis requires an unperturbed meiosis in germline cells during egg development (oogenesis) and a subsequent unchallenged mitosis in the embryo's soma. The repair of endogenous DNA DSBs induced by Mei-W68, a Spo11 homolog (McKim and Hayashi-Hagihara, 1998; McKim et al., 2002), is crucial for proper oogenesis. Meiotic DNA DSB repair produces inter-homolog recombination structures called crossovers that are essential for proper chromosome segregation. Proteins essential for recombinational repair of meiotic DSBs are also often required for mitotic break repair in somatic cells (Joyce et al., 2011; Lim and Hasty, 1996; Staeva-Vieira et al., 2003; Tsuzuki et al., 1996). Thus, defects in embryogenesis can originate from mutations in genes required for meiosis, mitosis, or both. Smc5/6 was described to act in both processes by resolving recombination products (Copsey et al., 2013; Gomez et al., 2013; Lilienthal et al., 2013; Xaver et al., 2013; Xue et al., 2014), however its function(s) within distinct cell division processes during the oocyte-to-embryo transition still remain elusive.

Here we generated *smc6* null flies, and along with a previously described *smc5* mutant by Li et al. (2013) we investigated the function of Smc5/6 during early *Drosophila* development. We discovered a maternal function of Smc5/6 in maintaining chromosome stability during both oogenesis and early embryogenesis. Smc5/6-deficient flies displayed female subfertility due to numerous mitotic defects in early embryos including degenerated nuclei and anaphase-bridge formation. Loss of Smc5/6 caused either a permanent arrest or delay in embryogenesis. Interestingly, early mitotic defects in *smc5/6* mutants during embryogenesis were linked to perturbed oogenesis. Although chromosome nondisjunction (NDJ) frequency and crossover rate were not greatly affected in *smc5/6* mutants, we observed persisting DSBs, a minor increase in X-chromosome nondisjunction (X-NDJ), and delayed pachytene progression. Surprisingly, the meiotic defects along with the early embryonic arrest of *smc5/6* mutants could be suppressed by lowering cultivation temperature for the females and hence oogenesis. The same suppression of meiotic defects could be seen when re-introducing a functional Smc6-6xHA transgene into a *smc6-*null background, which also led to diminished embryonic arrest. Thus, we suggest that Smc5/6 is required for proper embryogenesis in higher eukaryotes by maintaining genome integrity in both meiotic and somatic cells.

## RESULTS

### Mutants of Smc5/6 display reduced embryo viability

**Fig. 1.**
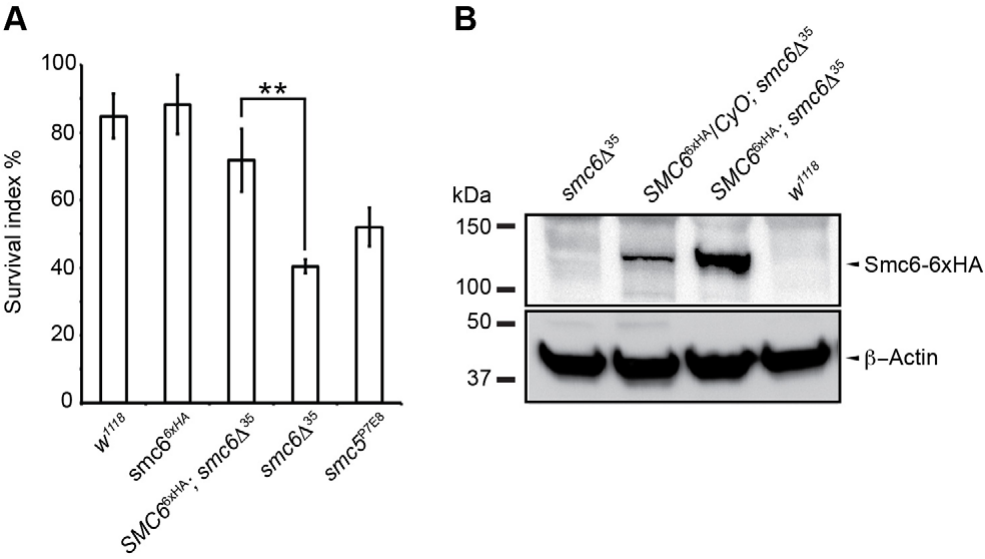
**Mutants of Smc5/6 display reduced egg viability.** (A) The egg-hatching frequency was determined as a measurement of embryo viability. Eggs were collected and incubated at 25°C. The total number of emerging flies was counted and plotted in percentage as a function of survival relative to wild-type. Each bar represents the mean of three independent experiments (*n*>500 eggs/experiment). Both *smc5*^P7E8^ and *smc6*Δ^35^ eggs revealed a 40% to 50% reduction in survival, respectively. The reduced survival rate of *smc6*Δ^35^ eggs was significantly suppressed by expressing the *SMC6*^6xHA^ transgene from chromosome 2 (**P=0.002). The statistical significance was determined with the Student's *t*-test. The error bars show standard variations. (B) Western blot analysis of embryonic crude extract (0-3 h) using a monoclonal HA-antibody confirmed the presence of Smc6-6xHA in embryos. Actin served as a loading control.

To study the function of Smc5/6 during embryogenesis we utilized a previously described loss-of-function allele *smc5*^P7E8^ (Li et al., 2013) (Fig. S1A). Moreover, we generated a *smc6-*null mutant, called *smc6*Δ^35^, by imprecise excision of an existing P-element, which caused a deletion of 1000 bp at the insertion site (Fig. S1A) (see Materials and Methods). Since mice *SMC6* knockouts were embryonic lethal, we examined the embryo viability of the *smc5/6* mutants. Embryo viability was investigated by egg-hatching rate. Eggs collected either from *smc5*^P7E8^ or *smc6*Δ^35^ females showed 40% to 50% reduction in hatching rate compared to wild-type (Fig. 1A). Furthermore, ectopic expression of a *SMC6*^6xHA^ transgene in the *smc6*Δ^35^ background rescued the hatch rate (Fig. 1A,B, Fig. S1A). This indicates that Smc5/6 is required for *Drosophila* embryogenesis.

### *Drosophila SMC5* and *SMC6* are maternal-effect genes

**Fig. 2.**
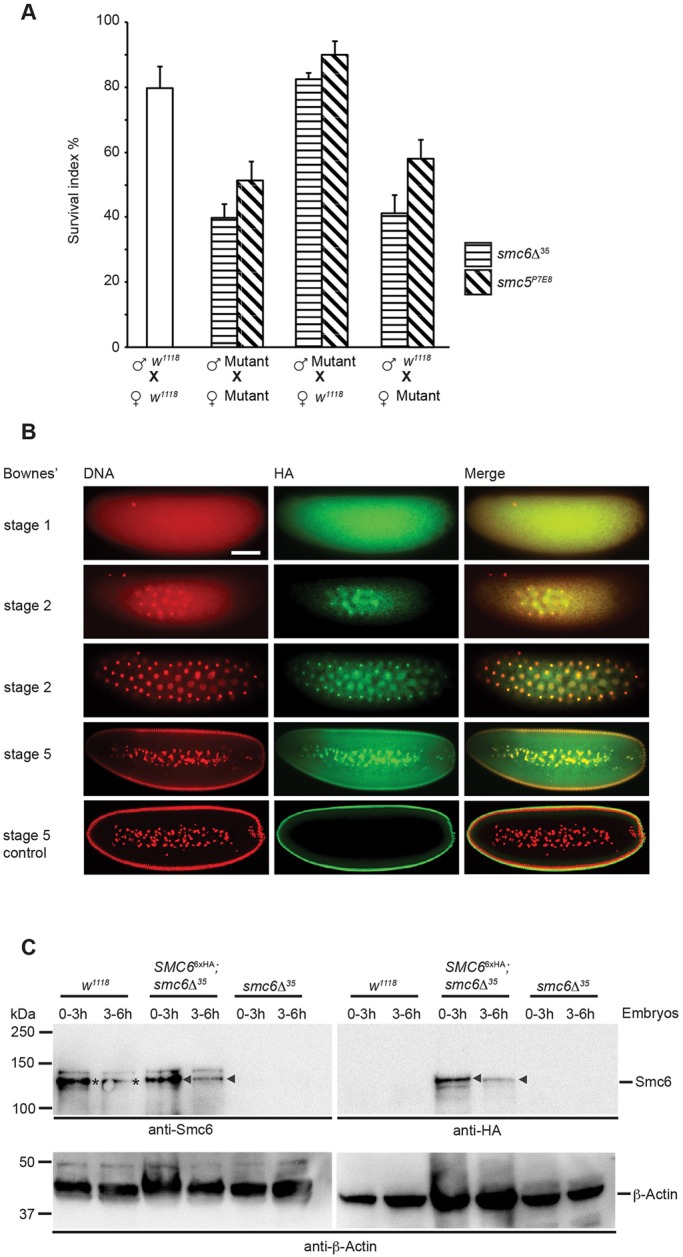
***Drosophila SMC5* and *SMC6* are maternal-effect genes.** (A) Hatching frequency of wild-type and *smc5/6* eggs. Mutant eggs fertilized by wild-type males showed a reduced hatch rate, whereas wild-type eggs fertilized by either *smc5*^P7E8^ or *smc6*Δ^35^ males did not. Each bar represents the mean of three independent experiments with standard deviations indicated (*n*>500 eggs/experiment). (B) Indirect immunofluorescence staining of early embryonic stages reveals binding of Smc6 to nuclei. DNA was stained with PI (red) and Smc6 localization was determined using the *SMC6^6xHA^* transgene in combination with a monoclonal HA-antibody (green). *w*^1118^ was stained as described and used as a negative control. Scale bar, 100 μm. (C) Western blot analysis of wild-type, *SMC6^6xHA^*, and *smc6*Δ^35^ embryonic crude extract from 0-3 and 3-6 h embryo collections. Asterisks indicate endogenous Smc6 levels and arrows indicate transgenic Smc6-6xHA levels, both of which were detected with an antibody raised against *Drosophila* Smc6. Transgenic Smc6-6xHA levels were also detected with a monoclonal HA-antibody against the HA-epitope tag. Actin served as a loading control.

Early embryogenesis in *Drosophila* lacks transcription of the zygotic genome. Instead inherited maternal products synthesized by the mother's genome drive early embryogenesis. Thus, reduced hatching frequency can originate from a gene defect within the mother's genome, known as a maternal-effect that subsequently affects the zygotic phenotype (Tadros and Lipshitz, 2009). We therefore investigated whether absence of Smc5/6 caused the reduced survival rate of *smc5*^P7E8^ and *smc6*Δ^35^ embryos. The hatching frequency was determined for wild-type, *smc5*^P7E8^, and *smc6*Δ^35^ eggs fertilized by either wild-type or *smc5/6* males. The egg-laying frequency of mutants and wild type were similar, which enabled the acquisition of an unbiased hatch rate (data not shown). From the reduced hatch rate of *smc5*^P7E8^ or *smc6*Δ^35^ eggs that were fertilized by wild-type males we concluded that Smc5 and Smc6 are maternally provided gene products (Fig. 2A). Furthermore, the hatch rate of wild-type eggs fertilized by either *smc5*^P7E8^ or *smc6*Δ^35^ males were not affected indicating that early embryonic developmental defects are solely caused by the maternal *smc5*^P7E8^ and *smc6*Δ^35^ alleles. Moreover, eggs from *smc5/6* females fertilized by wild-type males are naturally heterozygous for the *smc5*^P7E8^ or *smc6*Δ^35^ allele. This suggests that the reduced hatch rate is caused by early developmental defects at a time when the embryo still relies on maternally deposited products and the parental genome has not yet transcribed.

**Fig. 3.**
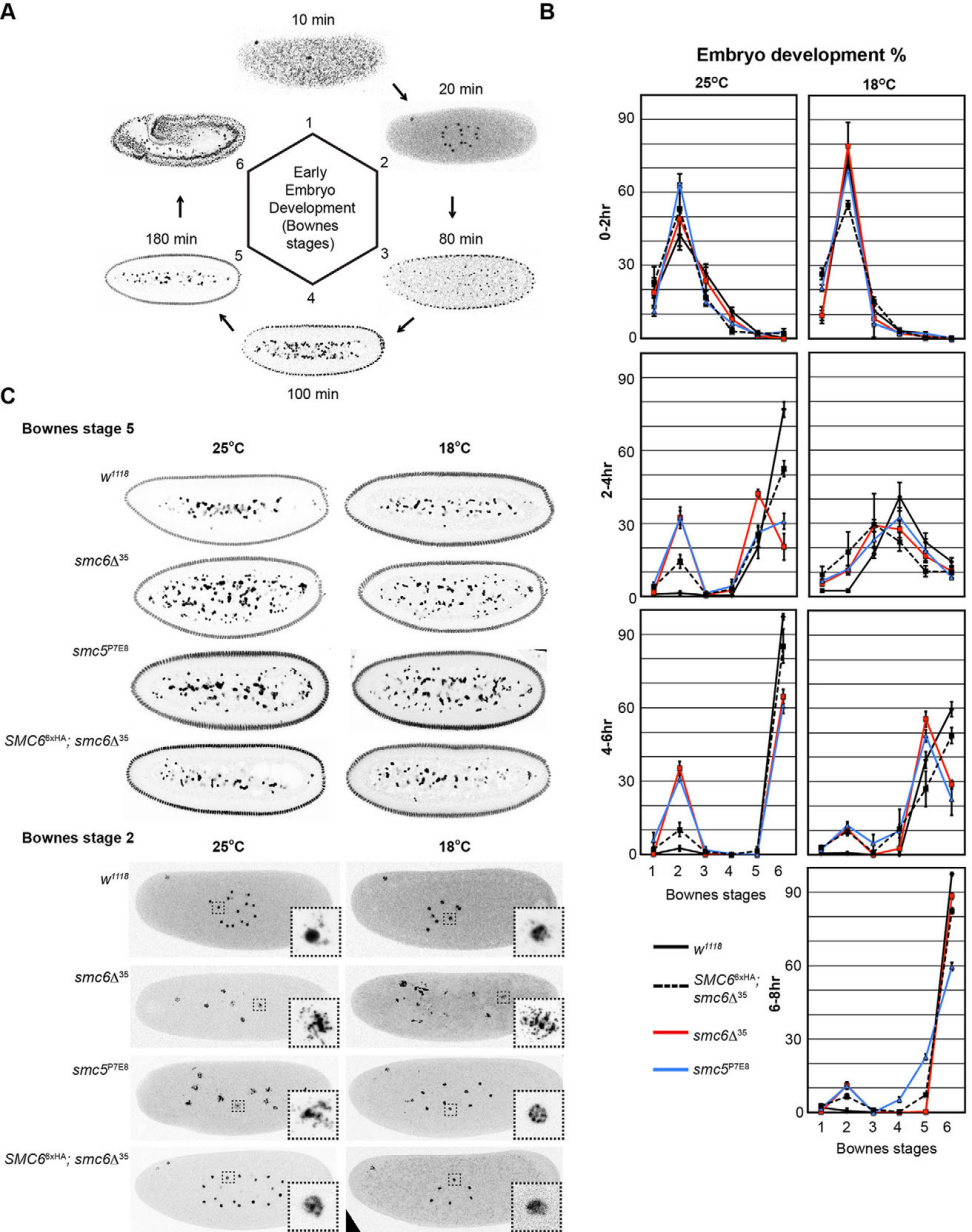
**Embryos of *smc5/6* mutants perform aberrant mitosis causing early and late embryonic defects.** (A) Overview of early embryogenesis including Bownes stages 1-6 after egg deposition. Embryos were fixed and DNA was stained with PI (grey). Timeline in minutes starts after egg deposition. (B) Embryos were collected from different time intervals and temperatures as indicated. Embryos were PI-stained and sorted according Bownes stage definitions. Each time-point represents the mean of three independent experiments with standard deviations indicated (*n*>500 for each time interval and experiment). At 25°C ∼35% of *smc5*^P7E8^ and *smc6*Δ^35^ embryos permanently arrested early at Bownes stage 2, whereas most mutant embryos that had reached Bownes stage 5 were delayed. At 18°C, embryogenesis progressed more slowly as expected, but led to a partial suppression of the early embryonic arrest from 35% to 10% for both *smc5*^P7E8^ and *smc6*Δ^35^. (C) Examples of wild-type, *SMC6*^6xHA^*; smc6*Δ^35^, *smc5*^P7E8^, and *smc6*Δ^35^ embryos with interphase nuclei are shown from Bownes stage 2 and 5. Embryos collected from different temperatures as indicated, were fixed and stained with PI (grey).

The role of maternally provided Smc6 was further explored by immunofluorescence staining of embryos (1-2 h old) from *SMC6*^6xHA^ transgene expressing females. Our custom-made polyclonal antibody against the N-terminal Smc6 region failed to detect endogenous Smc6. Therefore, an HA-antibody was used to detect Smc6-6xHA. Although Smc6-6xHA was expressed from an ectopic site, observations indicated that Smc6 transcription was still under a similar control compared to endogenous location. Immunofluorescence staining of polytene chromosomes from *SMC6*^6xHA^ transgene expressing larvae revealed absence of Smc6 (data not shown), which agreed with whole transcriptome RNA-sequencing data indicating that Smc6 is absent in polytene cells (Graveley et al., 2011). Conversely, microscopy analyses revealed Smc6 presence in early embryos (Fig. 2B), which western blot analysis confirmed using our custom-made Smc6 antibody (Fig. 3C). Smc6 was present in 0-3 h old embryos and was reduced in 3-6 h old embryos. Altogether we conclude that *SMC5* and *SMC6* belong to the class of maternal-effect genes, which have an early embryogenesis function.

### Smc5/6-deficient embryos exhibit mitotic defects during cleavage cycles

*Drosophila* embryogenesis have distinct developmental stages as described by Bownes (Bownes, 1975; Foe et al., 1993). Bownes' stages one to five include the first three hours of early embryogenesis after egg deposition that are largely dependent on maternal contribution of mRNA and proteins (Fig. 3A). After egg fertilization and within the first five Bownes' stages, the embryo undergoes 13 rapid nuclear division cycles starting with the zygotic nucleus. These divisions are known as cleavage cycles and occur in a common cytoplasm called the syncytium (Foe and Alberts, 1983).

To investigate early embryonic lethality of *smc5*^P7E8^ and *smc6*Δ^35^, we examined embryos collected at 25°C. Embryos were fixed, followed by propidium iodide (PI) staining and sorted by Bownes' stages (Foe et al., 1993). Two groups of mutant embryos with distinct developmental defects appeared during analysis. ∼35% of the mutant embryos collected from 2-4 h had arrested in Bownes' stage 2 instead of having reached Bownes' stages 4 to 6 (Fig. 3B), whereas mutant embryos that had reached Bownes' stage 5 were delayed before resuming embryogenesis. Confocal analyses of fixed embryos revealed distinct nuclear abnormalities. At Bownes' stage 2 most of the arrested mutant embryos exhibited a large number of degenerated nuclei within the yolk mass, whereas at stage 5 mutant embryos contained increased amounts of nuclear material spreading throughout the syncytium (Fig. 3C).

We next addressed whether the abnormal phenotypes of *smc5*^P7E8^ and *smc6*Δ^35^ embryos at Bownes' stage 2 and 5 reflected separate functions of Smc5/6 during embryogenesis. During the cleavage cycles approximately 1 h after egg deposition, the majority of the syncytial nuclei migrate to the embryos' cortex where they cellularize to form a monolayered epithelium that will provide the progenitors for all somatic tissues (Foe and Alberts, 1983). Notably, mitotic fidelity is sacrificed in favor of rapid proliferation, therefore *Drosophila* has evolved a Chk2-dependent process of ‘nuclear fallout’ that eliminates faulty nuclei from the embryo's cortex by actively removing them into the yolk (Iampietro et al., 2014; Sullivan et al., 1993b; Takada et al., 2003). Chk2 (*Drosophila* mnk) is a checkpoint kinase needed for cell cycle arrest and apoptosis by DNA damage.

**Fig. 4.**
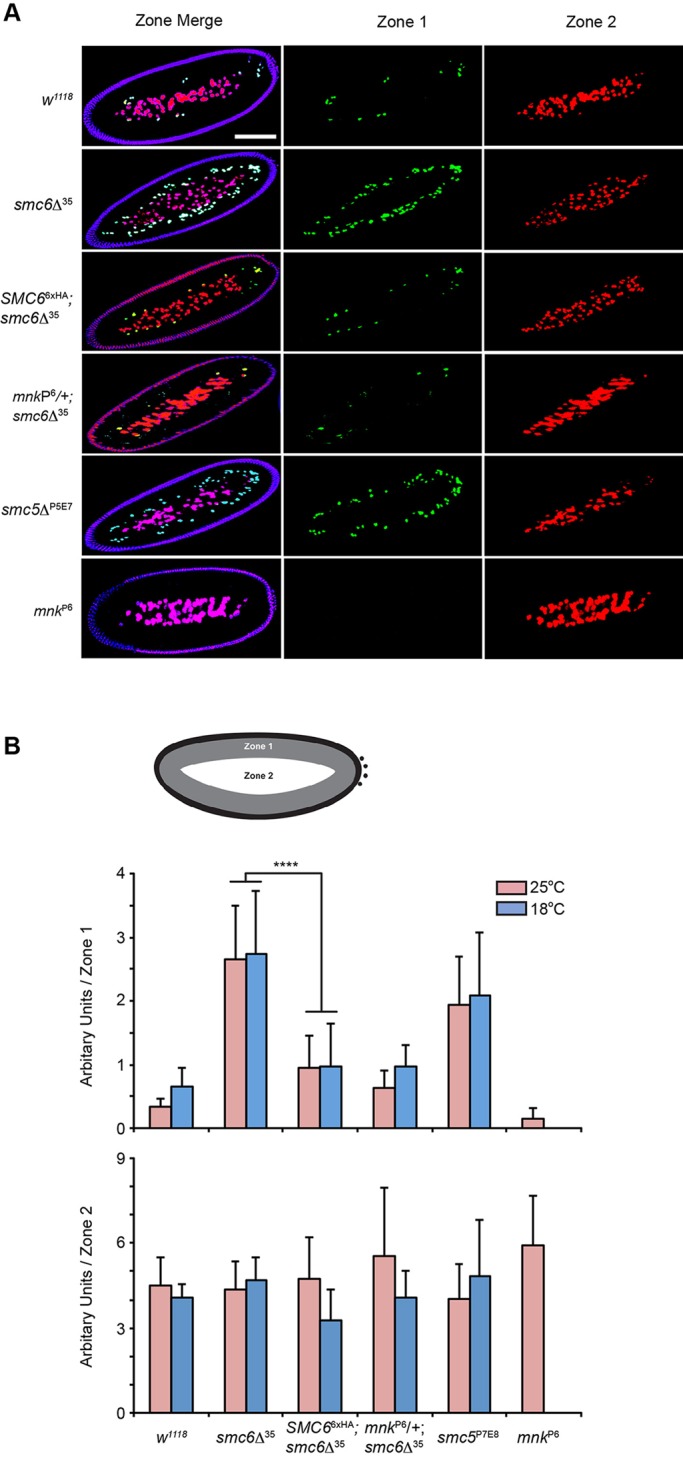
**Embryos of *smc5/6* mutants exhibit increased amount of Chk2-mediated nuclear fallout.** (A) Fixed and PI-stained embryos. Representative embryos are shown that are subdivided into zone 1 (green) and zone 2 (red). Each zone is highlighted with an artificial color. Scale bars, 100 μm. (B) Quantification of zone 1 and 2 from embryos (*n*=10) collected either at 18°C or 25°C. Embryos of *smc5*^P7E8^ and *smc6*Δ^35^ have increased, three- to fourfold, nuclear fallout within zone 1 regardless of temperature. *P*-values were determined for *smc6*Δ^35^ in comparison with the recue stock *SMC6*^6xHA^*; smc6*Δ^35^. ****P=3.5×10^−5^ at 25°C and ****P=2.3×10^−9^ at 18°C. The statistical significance was determined with the Student's *t*-test. The error bars show standard deviations.

In *smc5*^P7E8^ and *smc6*Δ^35^ embryos, multiple layers of nuclei beneath the cortex are observed which indicate erroneous nuclear divisions (Fig. 4A). By time-lapse microscopy of embryos expressing the His2Av-mRFP nuclear marker we visualized the ‘nuclear fallout’ in real-time. Unlike wild-type, nuclear fallout in *smc6*Δ^35^ embryos was enhanced by Bownes' stage 4 to 5 (Movies 1 and 2). We next quantified the intensity of damaged nuclei present as an estimate of nuclear division errors (see Materials and Methods). The embryos were subdivided into two zones after fixation and PI staining (Fig. 4B). Zone 1 encompassed the region of 20 μm beneath the surface, whereas zone 2 comprised the embryo's center including the yolk nuclei. Image analysis revealed that nuclear mass had increased three- to sixfold in zone 1 of *smc5*^P7E8^ and *smc6*Δ^35^, whereas in zone 2 the nuclear mass was similar in wild-type and mutant embryos (Fig. 4B). This suggests that early cleavage cycles are disrupted in *smc5/6* mutants causing an increase of damaged nuclei that will be eliminated from the cortex, which simultaneously delays gastrulation onset. Indeed a *mnk*^P6^/+; *smc6*Δ^35^ double mutant exhibited reduced nuclear fallout (Fig. 4A,B). Homozygous *mnk*^P6^ mutants served as a control, which lacked nuclear fallout; however due to the severe sickness of the homozygous *mnk*^P6^; *smc6*Δ^35^ double mutant, experiments were only permitted in a *mnk*^P6^ heterozygous background, most likely as a consequence from abolishing nuclear fallout and subsequent severe developmental defects.

**Fig. 5.**
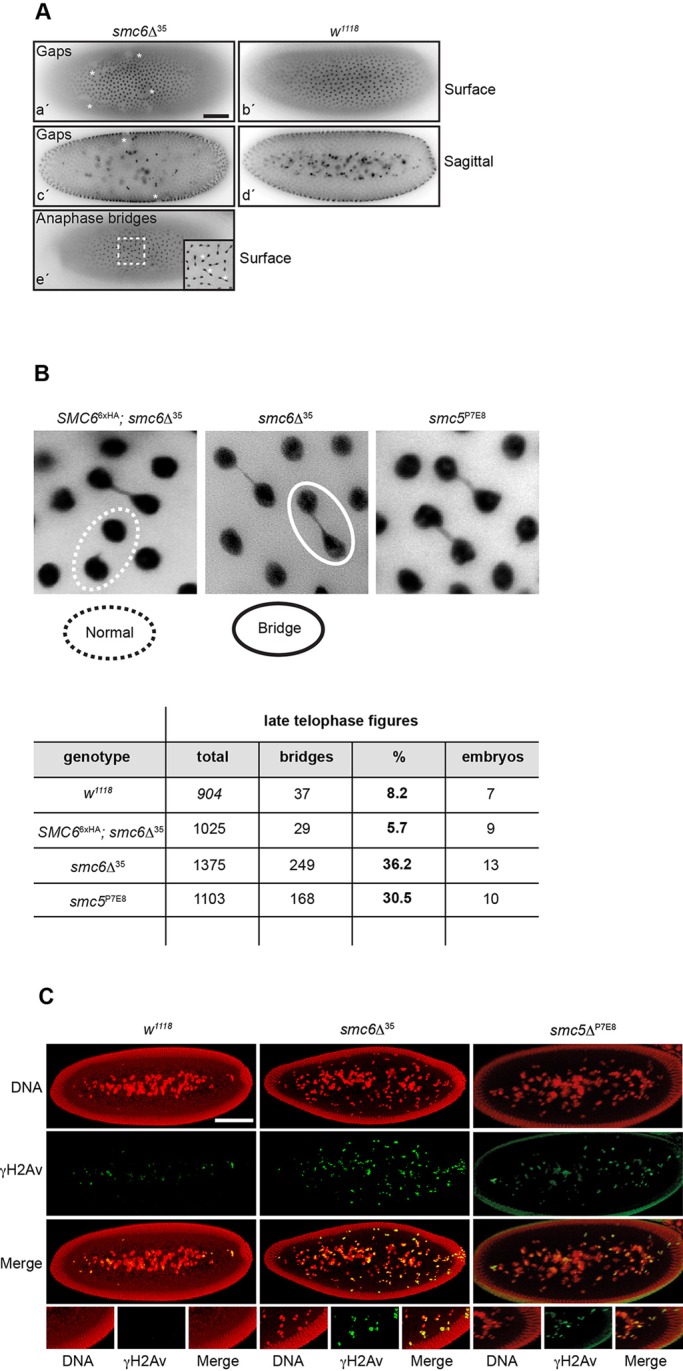
**Lack of maternal Smc5 and Smc6 induce anaphase-bridge formation in embryos.** (A) Fixed wild-type and *smc6*Δ^35^ embryo. DNA was PI-stained (grey). Embryos are represented in surface and sagittal view. In *smc6*Δ^35^ embryos (a′, c′, and e′) gaps (*) and anaphase bridges (*) are emphasized. Scale bar, 100 μm. (B) Quantification of anaphase bridges in *smc5/6* embryos. Embryos in late telophase were PI-stained (grey). Representative images with normal and bridging structures are shown. (C) Fixed wild-type, *smc5*^P7E8^, and *smc6*Δ^35^ embryos. DNA was PI-stained (red). The presence of DNA DSBs was detected with a monoclonal antibody against γ-H2Av (green). Embryos of *smc5*^P7E8^ and *smc6*Δ^35^ show increased accumulation of DNA DSBs beneath the surface. Scale bar, 100 μm.

Nuclear fallout results in gap formation in the uniform embryo's cortex. Thus, mutant embryos fixed after the cleavage cycles showed frequent gap- and anaphase-bridge-formation (Fig. 5A). Time-lapse microscopy of *smc6*Δ^35^ embryos revealed that anaphase bridged nuclei were eliminated and simultaneously left a gap (Movie 3). To quantify DNA-bridging frequency, fixed embryos were examined at late telophase (nuclear cycle 10-13) (Fig. 5B). The DNA-bridging frequency in *smc5/6* embryos was ∼fourfold higher than in wild type, suggesting that lack of maternal Smc5 or Smc6 causes chromosome segregation defects.

Moreover, DNA damage mediated under genotoxic conditions has been suggested to induce nuclear fallout (Sibon et al., 2000; Sullivan et al., 1993a). We therefore investigated the presence of phosphorylated γ-H2Av, which marks DSBs (Lake et al., 2013). In *smc5*^P7E8^ and *smc6*Δ^35^ embryos, γ-H2Av foci accumulated more than in wild type (Fig. 5C). Interestingly most γ-H2Av foci appeared beneath the surface, suggesting that the enhanced nuclear fallout seen in *smc5*^P7E8^ and *smc6*Δ^35^ embryos may not be caused by increased DNA DSB at the cortex. Instead, this may be an unknown mechanism that targets abnormal nuclei for degradation by introducing DNA DSBs upon their fallout.

### Reduced developmental growth rate suppresses early mitotic arrest of *smc5/6* embryos

**Fig. 6.**
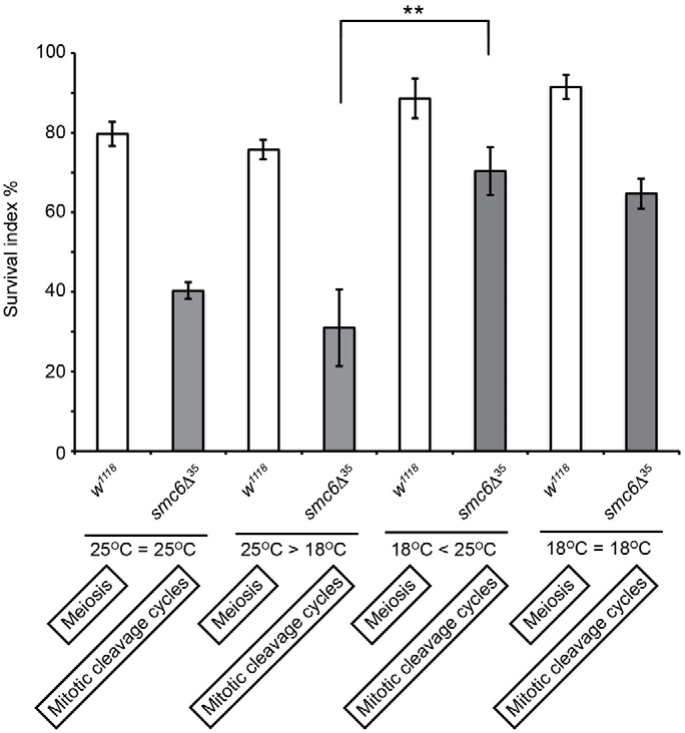
**Low temperature during oogenesis increases the hatching frequency of *smc6* eggs.** Wild-type and *smc6*Δ^35^ eggs were collected either at 18°C or 25°C followed by incubation at 18°C or 25°C, and vice versa for further development until hatching. The female cultivation temperature affects oogenesis, whereas the subsequent egg incubation affects embryogenesis. The total number of emerging flies was counted and plotted in percentage as a function of survival relative to wild-type. Each bar represents the mean of three independent experiments (*n*>500 eggs/experiment). Eggs of *smc6*Δ^35^ females collected at 18°C showed increased hatch rate compared to eggs collected at 25°C (**P=1.6×10^−3^). The subsequent developmental temperature during the mitotic cleavage cycles had no impact on the hatch rate. The statistical significance was determined with the Student's *t*-test. The error bars show standard deviations.

In bacteria, *Bacillus subtilis*, chromosomal defects caused by deletions of SMC subunits were suppressed under slow growth conditions that reduce replication fork velocity (Gruber et al., 2014). Therefore, we tested whether the nuclear division defects in *smc5/6* embryos were suppressible by reducing the rapid progression of the cleavage cycles; to slow down the cleavage cycles we modified the cultivation temperature (Kuntz and Eisen, 2014). Embryos incubated at 18°C were collected from different time intervals and examined to determine their developmental stage. As expected, most embryos delayed their development at 18°C compared with embryogenesis at 25°C, which was evident among embryos collected from a 2-4 h time interval (Fig. 3B). However, the nuclear fallout frequency in *smc5/6* embryos at Bownes' stage 5 was not suppressed by a temperature shift (Fig. 4B), but instead we observed a suppression of Bownes' stage 2 arrested embryos (Fig. 3B). We confirmed this by a systematic embryo collection, which distinguished whether the temperature effect was mediated through a change in meiotic behavior or an increase in mitotic fidelity. To address whether low temperature could change the meiotic behavior of *smc5/6* oocytes, we collected eggs from *smc6*Δ^35^ females at 18°C and incubated them at 25°C (or vice-versa) for further development until hatching (see Materials and Methods). Interestingly eggs from *smc6*Δ^35^ females cultivated at 18°C, as opposed to 25°C, showed an improved hatch rate (Fig. 6), thus in *smc6*Δ^35^ embryos early mitotic defects are likely caused by errors during female meiosis that are suppressed by low temperature, which we will refer to as temperature-shift phenomenon.

### Smc6 localizes to the oocyte during oogenesis

**Fig. 7.**
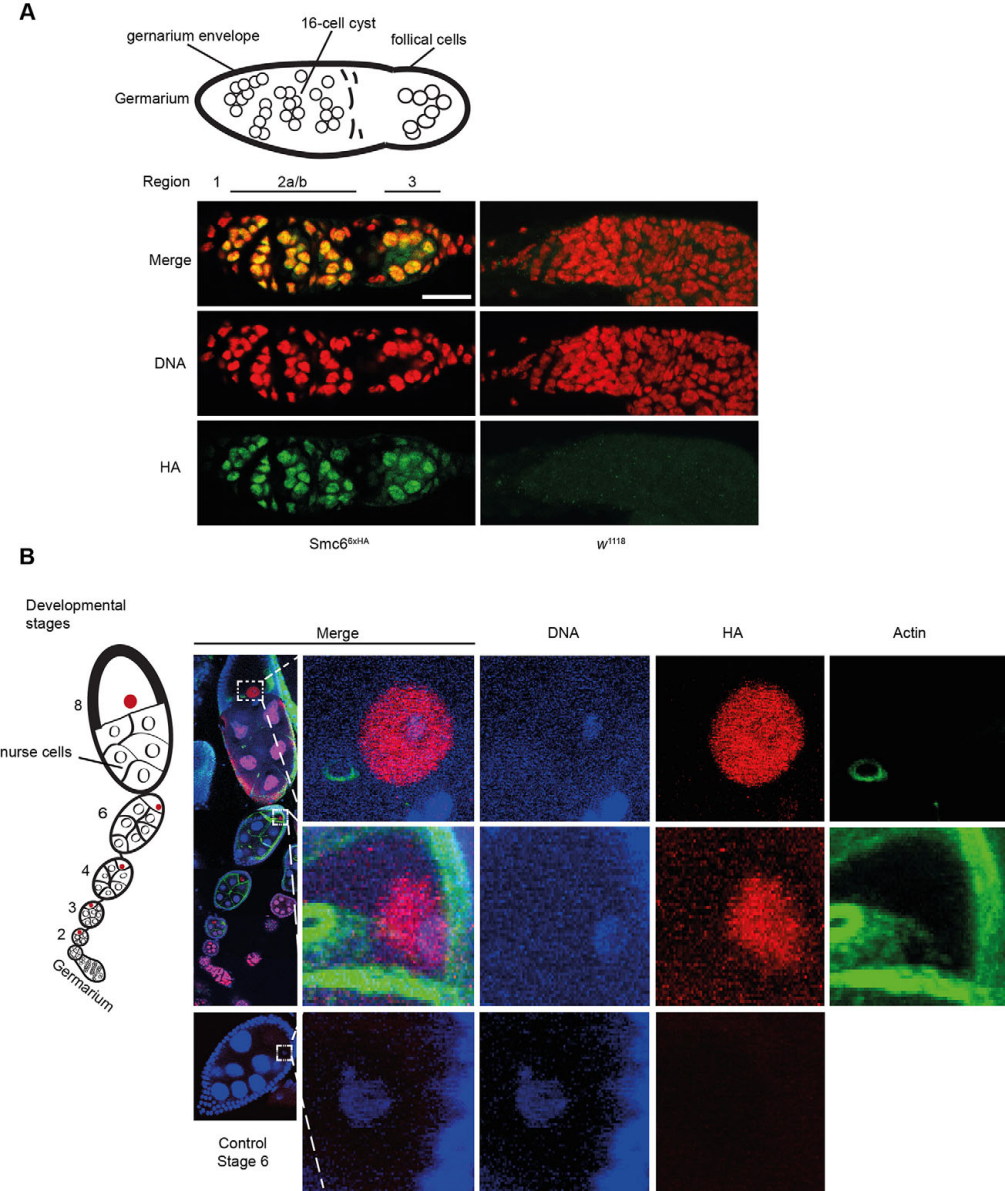
**Smc6 localization during oogenesis.** The localization of Smc6 in ovaries was determined using the *SMC6*^6xHA^ transgene in combination with a monoclonal antibody against the HA epitope-tag. *w*^1118^ was stained and served as a negative control for the HA-antibody. Ovarioles (drawing on left side in figure B represents one ovariole) were dissected, fixed, and stained. DNA staining in red and Smc6 staining in green. (A) Smc6 localizes to the germarium within the mitotic and meiotic germline cysts. Scale bar, 5 μm. (B) Projected *z*-stacks of confocal images taken with 63× objective that have been stitched together to fit an ovariole. DNA stain in blue, Smc6 in red, and actin in green. Smc6 localizes to the karyosome and germinal vesicle (see stage 8). Smc6 also localizes to nurse cells up to stage 3, whereby it disappears and re-appears at nurse cells again at stage 8.

*Drosophila* ovaries consist of 16 to 20 ovarioles that each contains a linear array of oocytes of different developmental stages, whereby meiosis initiates in the ovarioles most anterior part called the germarium (Fig. 7) (Ogienko et al., 2007; Spradling et al., 1997). The germarium is subdivided into four regions (1, 2a, 2b, and 3) representing different developmental stages of pre-meiotic and meiotic oogenesis. In region 1, mitotic cell divisions produce a 16-cell cyst from a germline stem cell. In region 2a and 2b the 16-cell cyst enters the meiotic program, which is accompanied with synaptonemal complex assembly, DNA DSB formation, and meiotic recombination. When the cyst proceeds to region 2b, the oocyte is determined from one of the 16 cells and the remaining cells develop into nurse cells. In region 3, the oocyte localizes to the posterior with no DNA DSBs, which marks the start of egg chamber development.

To define the role of Smc6 in meiosis, we used an anti-HA antibody to examine Smc6-6xHA localization in ovaries from transgenic flies. In the ovariole, Smc6 was visible throughout all four stages of the germarium (Fig. 7A). Here, Smc6 was present within mitotic and meiotic germline cysts, but excluded from other somatic germarial cells such as follicle cells. Interestingly, Smc6 localized to the oocyte nucleus throughout all visible stages of oogenesis; however at stage 8 Smc6 was most apparent in the germline vesicle and on the condensed oocyte nucleus known as the karyosome (Fig. 7B; Spradling et al., 1997). Moreover at stage 2 of oogenesis, Smc6 also localized to the nuclei of all nurse cells until stage 3, whereby Smc6 staining became diffuse and not detectable in nurse cells until stage 8.

### Meiotic absence of Smc5/6 increases X-NDJ

Proteins that preferably localize to the germline nuclei of the germarium are usually involved in pairing of homologous chromosomes and/or meiotic recombination. Smc6 has been implicated in recombinational repair and meiosis (Lilienthal et al., 2013), we therefore addressed whether *smc5/6* mutants interfere with the meiotic recombination process in *Drosophila* females. We measured X-chromosome nondisjunction (X-NDJ) frequency by mating *smc5*^P7E8^ and *smc6*Δ^35^ females to males. The male sperm contained compound chromosome X^Y carrying a dominant marker ***B*** that effected eye shape and allowed us to measure X-NDJ frequency (see Materials and Methods). We measured a NDJ frequency of 2.45% and 1.66% for *smc5*^P7E8^ and *smc6*Δ^35^, respectively, which was six- to 17-fold increased compared to wild-type females (Table 1). Thus, we suggest that meiotic recombination is affected in *smc5/6* females causing improper chromosome segregation during meiosis. Furthermore, NDJ-frequency was reduced by three- to fivefold when cultivating *smc5/6* flies at 18°C. This is in line with the temperature-shift phenomenon. We also investigated if the autosomal chromosomes 3 and 4 underwent NDJ in *smc6*Δ^35^ females (Table S1 and Material and Methods). Surprisingly, neither autosomal chromosomes exhibited a difference in NDJ-frequency between *smc6*Δ^35^ and wild-type females suggesting that Smc5/6 is mainly required for X-chromosome segregation during meiosis. Normally, increased meiotic nondisjunction arises from recombination defects along with abolished or reduced crossovers (Grell, 1979), we therefore tested X*-*chromosome crossover rate (Fig. S2 and Materials and Methods). Surprisingly, we found that crossover rate was not severely changed suggesting that increased X*-*NDJ frequency in *smc5/6* females is not due to an altered crossover rate.

**Table 1.**
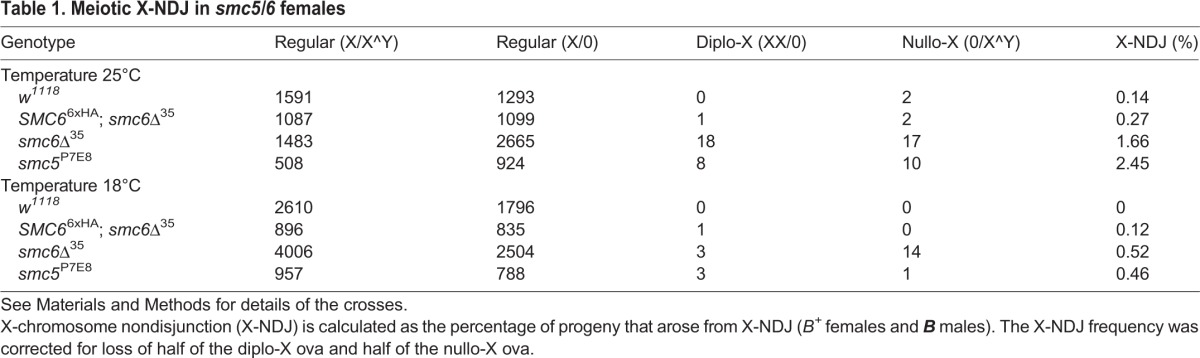
**Meiotic X-NDJ in *smc5/6* females**

### Meiotic DNA DSB repair is insufficient in *smc5/6* ovaries

**Fig. 8.**
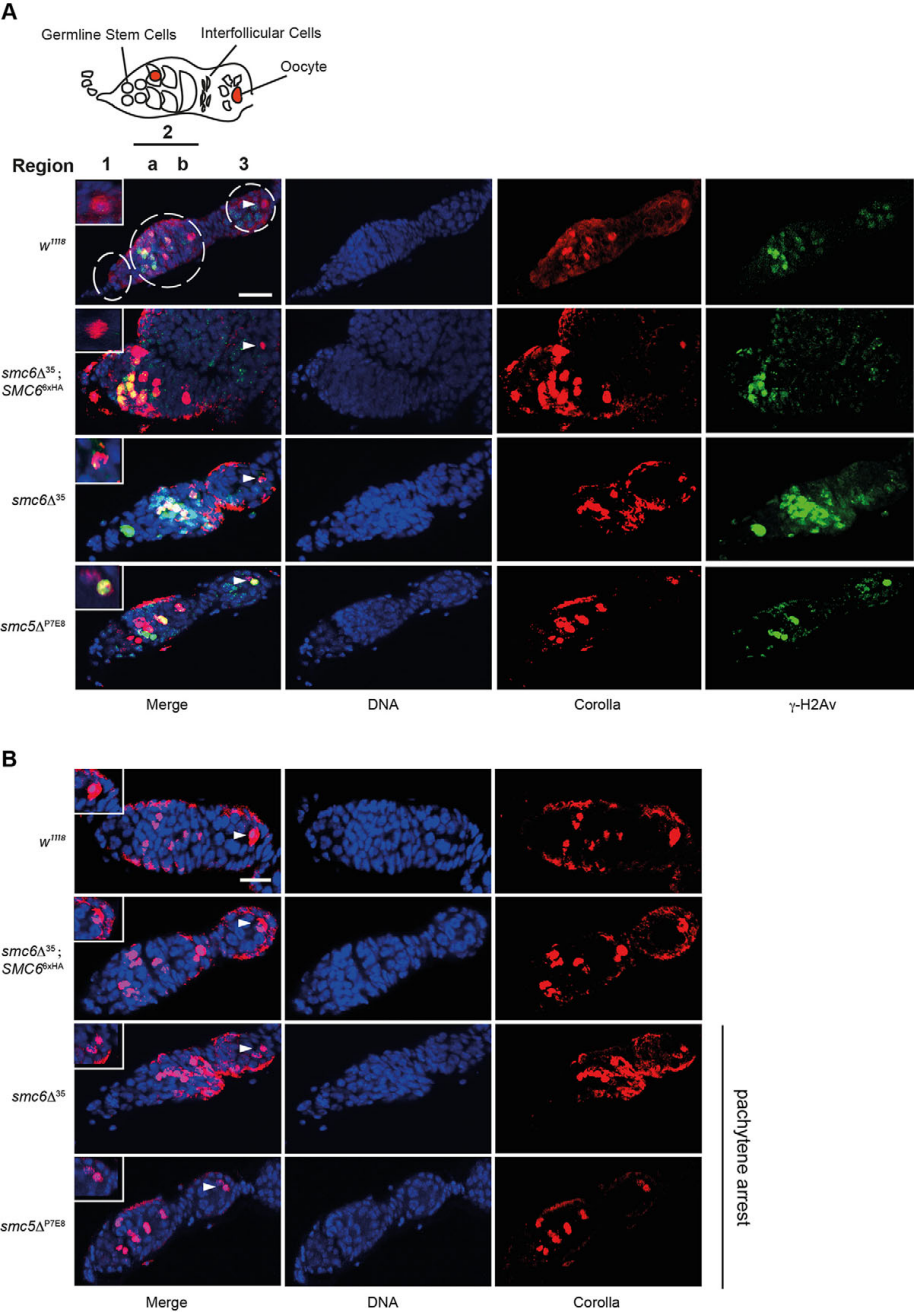
**Mutants of *smc5/6* exhibit DNA DSB formation and pachytene arrest.** Images show representative *z*-stack projections of germaria from wild-type, *SMC6*^6xHA^; *smc6*Δ^35^, *smc6*Δ^35^, and *smc5*Δ^P7E8^ (only images from 25°C experiments are shown). DNA is stained in blue, corolla in red, and γ-H2Av foci in green. Oocytes in region 3 are marked with an arrowhead. (A) In *smc5/6* mutants, DSBs remain in region 3, which co-localize with the posterior positioned oocyte. Scale bar, 10 μm. (B) Germaria with either normal meiotic progression (*w^1118^* and *SMC6*^6xHA^; *smc6*Δ^35^) or pachytene arrest (*smc6*Δ^35^ and *smc5*Δ^P7E8^). Pachytene arrest is indicated by the presence of two oocytes in region 3 (arrowhead). Scale bar, 5 µm.

In the germaria, meiotic DSBs are induced in region 2a and repaired before the oocyte enters region 3 (Jang et al., 2003). Genes required for DSB repair in meiotic germline cells encode homologs of the Rad52 epistasis group (Joyce and McKim, 2011). Mutations in these genes cause an accumulation of γ-H2Av foci that persist throughout prophase and represent unrepaired meiotic DSBs (Staeva-Vieira et al., 2003). We investigated whether increased NDJ-frequency reflected defective meiotic DSB repair during prophase by examining the kinetics of γ-H2Av foci in the germaria of *smc5*^P7E8^ and *smc6*Δ^35^ females. Since meiotic DSBs are initiated at random among all 16 cyst-cells, we used an anti-corolla antibody to distinguish the pro-oocytes such that DSBs were only counted in corolla-stained nuclei of regions 2a, 2b, and 3 (see Material and Methods) (Mehrotra and McKim, 2006). Corolla is a transverse filament protein of the central region in the synaptonemal complex and has been used to mark pro-oocytes (Collins et al., 2014). Surprisingly, examining the number of γ-H2Av foci in germaria revealed similar kinetics in wild-type and *smc5/6* mutants (Fig. 8A; Table S3, Fig. S3). Moreover, no DSBs were detected in oocytes of *mei-W68*^2-0949^ confirming that γ-H2Av foci were dependent on Mei-W68 induced DSB formation. Intriguingly, however, a few DSBs were present in *mei-W68*^2-0949^; *smc6*Δ^35^ double mutants in some of the oocytes. This likely represented the mitotic breaks generated during the pre-meiotic cell divisions (Table S3, Fig. S3). Notably, even though the number of DSBs in region 3 oocytes was similar to wild type, we observed many *smc5/6* oocytes within germarial region 3 containing few persisting DSBs. Thus, due to the incomplete penetrance of the mutant phenotype, we examined the frequency of these oocytes and found a two- to threefold increase compared to wild type suggesting that meiotic and/or mitotic DNA DSBs are insufficiently repaired in *smc5/6* mutants (Table 2). This frequency decreased when fly stocks were cultivated at 18°C instead of 25°C. This concurred with the observed temperature-shift phenomenon and suggests that disturbances during oogenesis can proceed into embryogenesis. Notably, the rescue stock *SMC6*^6xHA^; *smc6Δ*^35^ also diminished the frequency of oocytes with remaining DSBs.

**Table 2.**
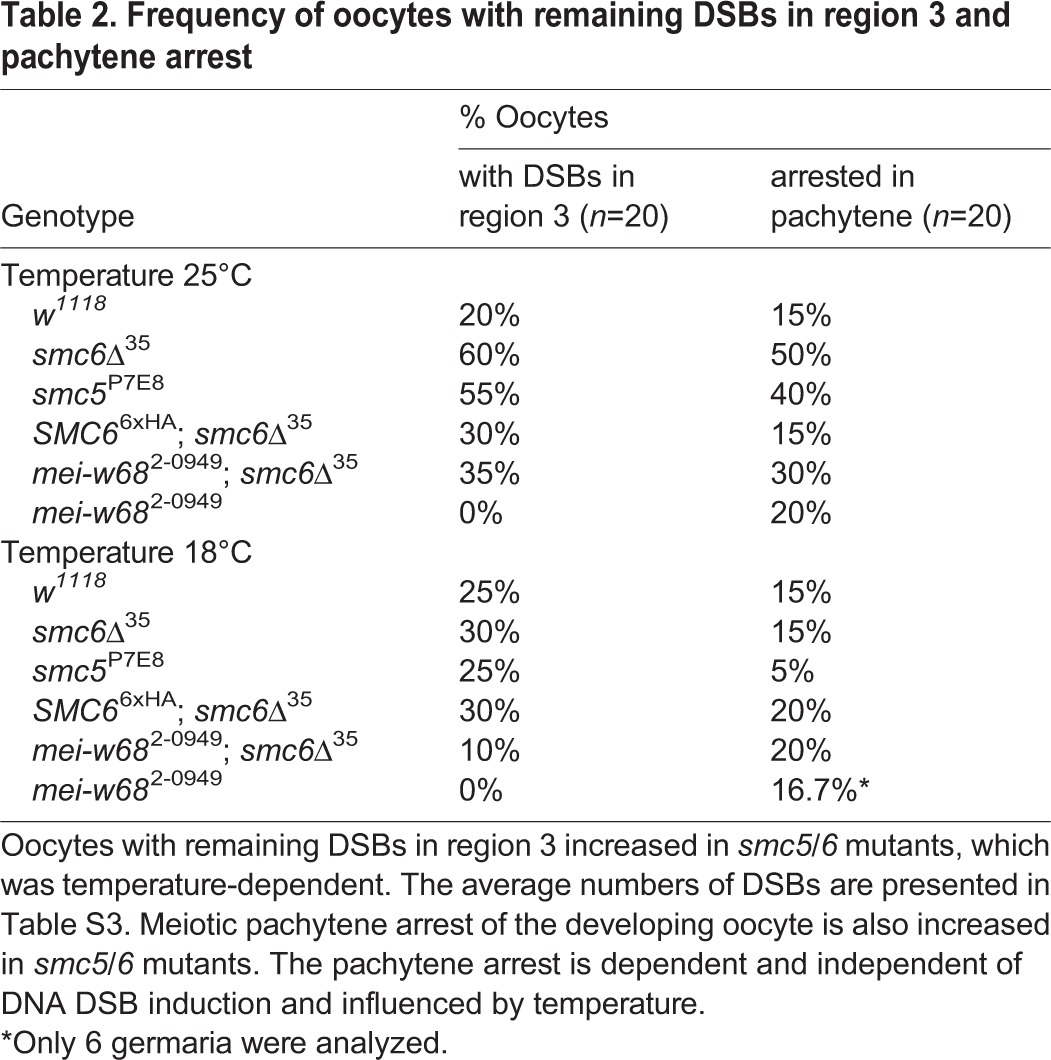
**Frequency of oocytes with remaining DSBs in region 3 and pachytene arrest**

Persisting DSBs trigger Mei-41- (*Drosophila* ATM homolog) dependent DSB repair checkpoint activation that delays oogenesis (Joyce and McKim, 2011). In DNA repair-deficient mutants, this checkpoint activation leads to an abnormal eggshell phenotype, which manifests as a dorsal-ventral polarity defect in embryogenesis (Ghabrial and Schupbach, 1999; Gonzalez-Reyes et al., 1997; Staeva-Vieira et al., 2003). Furthermore, the embryo polarity is established in oogenesis and is dependent on the localization and translation of *gurken* mRNA, which is disrupted in DNA repair mutants. However, in *smc5/6* mutants, no altered patterning of the eggshell or distribution of Gurken protein was observed (data not shown and Fig. S4), suggesting that the few persisting DSBs in *smc5/6* mutants do not activate the DNA damage checkpoint.

### Mutants of Smc5/6 undergo pachytene arrest and have delayed oocyte chromatin (karyosome) dynamics

To further investigate the meiotic behavior of *smc5/6* mutants we examined oocyte differentiation through meiotic pachytene progression. In region 3, oocyte determination should have finalized along with posterior positioning. The meiotic progression in *smc5/6* mutants was examined by corolla staining. In both *smc5*^P7E8^ and *smc6*Δ^35^ ovaries, oocyte determination was frequently delayed. Quantification revealed a frequency of 40% and 50% of two corolla-stained oocytes in region 3 in *smc5*^P7E8^ and *smc6*Δ^35^ germaria, respectively (Table 2, Fig. 8B). This phenomenon is called pachytene arrest and was described for DSB repair and exchange mutants, as a DSB-independent pachytene checkpoint (Joyce and McKim, 2009). Interestingly, in this study, pachytene arrest was decreased in a *mei-W68*^2-0949^; *smc6*Δ^35^ double mutant suggesting that meiotic DSBs may impair the recovery from the checkpoint, or perhaps play a role in pachytene checkpoint activation. Moreover, the delay in pachytene progression was suppressed by cultivating the fly stocks at 18°C instead of 25°C. The same suppression was also seen for the rescue stock. Thus, pachytene checkpoint activation is influenced by both temperature and persisting DSBs in *smc5/6* mutants.

We extended the analysis of meiotic progression in *smc5/6* mutants by investigating the oocyte nucleus at late- and post-pachytene stage. Here we discovered that karyosome dynamics were delayed in *smc5/6* mutants, which were averted in a *mei-W68*^2-0949^; *smc6*Δ^35^ double mutant, by lowering the cultivation temperature, or in the presence of a functional *SMC6*^6xHA^ transgene (Fig. S5,S6). Thus, we suggest that DSBs delay oogenesis also in aspects other than oocyte selection.

## DISCUSSION

Smc proteins are essential in most organisms for cell viability and genome integrity. Functional developmental studies are usually limited to less ideal conditional mutants. In *Drosophila*, *smc5/6* null mutants are moderately viable and hence suitable for developmental studies. Here we investigated *smc5/6* flies and observed a maternal function of Smc5/6 in maintenance of chromosome stability during early embryogenesis. Embryos from mutant females showed various nuclear defects prior to gastrulation including degenerated nuclei and increased formation of anaphase bridges and DNA DSBs. Furthermore, Smc5/6-deficient flies displayed female subfertility, which was shown by reduced hatch rates of *smc5/6* eggs. We believe that this phenotype stems from perturbations during meiosis and oogenesis that proceed into embryogenesis. Indeed, *smc5/6* mutants displayed several defects in ovarian germline cells including unresolved mitotic and meiotic DNA DSBs, increased X-NDJ, and delayed pachytene progression and karyosome dynamics.

During early embryogenesis *smc5/6* embryos exhibited numerous mitotic defects that delayed gastrulation onset. We propose that increased anaphase-bridge formation caused the delay by prolonging nuclear fallout duration. Nuclear fallout is a Chk2 (mnk)-dependent process that removes damaged nuclei from the cortex to prevent the inclusion of damaged precursor cells. The *mnk*^P6^ single mutant lacked nuclear fallout while being highly viable, whereas the *mnk*^P6^; *smc6*Δ^35^ double mutant had almost no progeny. This suggested that the unique ability to perform nuclear fallout partly allows *Drosophila* to escape Smc5/6 essentialness.

The enormous activation of DNA replication origins during the cleavage cycles in early *Drosophila* embryogenesis (Blumenthal et al., 1974; McKnight and Miller, 1977; Spradling and Orr-Weaver, 1987) can cause DNA fork perturbations known to generate regions of unreplicated DNA, consequently anaphase-bridge formation takes place and ultimately DNA breakage (Sofueva et al., 2011). Based on published data showing a requirement of Smc5/6 for fork stability during DNA replication (Irmisch et al., 2009; Kegel et al., 2011; Xue et al., 2014), we believe that anaphase bridges occur frequently in *smc5*^P7E8^ and *smc6*Δ^35^ embryos as the cleavage cycles induce replication stress by using many origins of replication. However, we cannot exclude that other additional functions of Smc5/6 during the cleavage cycles cause nuclear fallout. A link between yeast Smc5/6 and the mitotic spindle was recently shown whereby Smc5 interacted with microtubules and a mutation within the microtubule-binding region of Smc5 caused aberrant chromosome segregation and spindle structure (Laflamme et al., 2014), thus the many functions of Smc5/6 in genome integrity appear complex and cannot yet be individually assigned to each aberrant development phenotype observed.

Apart from Bownes stage 5 delay, *smc5/6* embryos also encountered problems during the initial mitotic cleavage cycles manifesting in an early arrest in embryogenesis (Bownes stage 2). Together the early embryonic arrest correlated with the observed 40%-50% reduced hatch rate in *smc5/6*. Although a recent study by Li et al.(2013) implied only a minor reduction in hatch rate, in this study their *smc5*^P5E7^ along with our *smc6*Δ^35^ mutant exhibited a more severe phenotype during embryogenesis (Li et al., 2013). This is in line with a previous study, where the Nse3 homolog was depleted in *Drosophila* and Smc5/6 was suggested to be essential for embryogenesis (Nishimura et al., 2008). Additionally, early mitotic fidelity in *smc5/6* embryos improved when subjecting oogenesis to a temperature-shift. The survival rate of *smc5/6* eggs increased by propagating oogenesis at a low temperature (18°C), suggesting that Smc5/6 has a vital meiotic function at high ambient temperatures. Temperature influence over meiosis was previously recognized, whereby chromosome form and behavior were different upon temperature change causing altered internal coiling, frequency, and positioning of crossovers (Plough, 1917). Accordingly, by cultivating *smc5/6* female flies at 18°C instead of 25°C we observed suppressed meiotic defects, which included unresolved DNA DSBs, X-NDJ, and delay in pachytene progression and karyosome dynamics. The same suppression was seen for the *SMC6*^6xHA^ transgene in a *smc6*Δ^35^ background. Since both temperature-shift and the transgene increased embryonic viability, we suggest that decreasing meiotic defects in oogenesis can improve progression of embryonic development.

In yeast, *smc5/6* mutants accumulate recombination intermediates, known as joint molecules (JMs). These form between sister chromatids instead of inter-homologs and inhibit accurate chromosome segregation (Lilienthal et al., 2013). Sister-chromatid JMs require resolution before metaphase II to prevent chromosome nondisjunction. Notably, measuring X*-*NDJ frequency of *Drosophila smc5/6* females revealed a six- to 17-fold increase compared to wild type, however the overall frequency is relatively low compared to previously described NDJ mutants (Giunta et al., 2002). Surprisingly only the X-chromosome was affected, whereas the autosome 3 and achiasmate chromosome 4 segregated normally. This could potentially be attributable to the ribosomal DNA (rDNA) clusters retained solely on *Drosophila* X-chromosome. Since rDNA consist of repetitive sequences, HR-dependent DNA damage repair could induce illegitimate recombination events that generate JMs and unequal sister chromatid exchange (Eckert-Boulet and Lisby, 2009). Indeed, *smc5/6* mutants of *Saccharomyces cerevisiae* cause rDNA instability and missegregation indicating a crucial function for Smc5/6 in its maintenance (Torres-Rosell et al., 2005). However, due to the low number of X*-*NDJs in *smc5/6* mutants compared to canonical NDJ mutants we did not examine persisting JM structures in meiosis II, which we believe would not be abundant enough to detect.

Efficient DSB repair via HR is essential for proper chromosome segregation. We tested the efficiency of meiotic DSBs break repair in *smc5/6* mutants with regard to the increased X-NDJ rate. Meiotic DSB repair as monitored by γ-H2Av foci dynamics revealed similar kinetics in wild-type and *smc5/6* mutants; however we observed a substantial presence of oocytes in region 3 with persisting DSBs in *smc5/6* mutants as compared to wild-type. This is in line with *smc5/6* mutants being defective in DSB repair. Interestingly, persisting DSBs were not completely abolished in a *mei-W68*^2-0949^; *smc6*Δ^35^ double mutant indicating that unresolved DSBs reflect impaired break repair during both meiosis and the mitotic cycles of cyst development. Thus, we propose that the low, but yet increased X*-*NDJ-frequency is caused by the few persisting DSBs found in *smc5/6* mutants. Increased NDJ would be expected to interfere with proper chromosome segregation and hence reduce meiotic recombination, however crossover rates in *smc6*Δ^35^ females were unaffected, which is not surprising based on similar findings from already published yeast data (Lilienthal et al., 2013)*.* At the moment we cannot explain the relationship between X*-*NDJ and crossover rates observed here, but due to the low X-NDJ-frequency in *smc5/6* mutants alteration in crossover rates might not be apparent; thus the role of Smc5/6 during oogenesis appears complex. Smc5/6 may have a dual function in pre-meiotic DNA replication and meiotic DSB break repair, which are prerequisites for the establishment of proper meiotic recombination and oocyte maturation.

A shared feature by *smc5/6* and DNA repair defective mutants is their impact on oocyte determination and nucleus morphology. In both DNA repair and *smc5/6* mutants, oocyte determination is delayed by pachytene checkpoint activation (Joyce and McKim, 2009). Furthermore, in DNA repair mutants the oocyte chromatin is less organized and diffuse at late pachytene stages (Staeva-Vieira et al., 2003). In comparison to wild-type, *smc5/6* mutants exhibited a delay in compaction during pachytene followed by a delay in relaxation at post-pachytene stages. The difference between DNA repair and *smc5/6* mutants in karyosome formation might be explained by number of DNA DSBs. In DNA repair mutants, numerous γ-H2Av foci accumulate in the oocyte nucleus that eventually perturb the karyosome organization, whereas the few persisting DSBs in *smc5/6* oocytes might be tolerated. Interestingly, a *mei-W68*^2-0949^; *smc6*Δ^35^ double mutant partially suppressed pachytene arrest frequency and altered karyosome dynamics, indicating that unresolved DSBs contribute to a delay in oogenesis progression. Conversely, others showed via a *mei-W68* single mutant that pachytene arrest is not dependent on DSB-induction or persistent repair intermediates (Joyce and McKim, 2009), which we also see when using a *mei-W68*^2-0949^ single mutant. This precarious readout, however, may be attributable to persisting DSBs impairing Smc5/6 function in mitosis, meiosis, and potentially additional roles in chromosome organization and structure. Indeed Smc4, which is part of the condensin complex, also localizes to the oocyte chromosomes indicating that Smc proteins play a central role in karyosome formation and maintenance (Ivanovska et al., 2005).

DNA repair mutants commonly have an altered patterning of the eggshell, which manifests as a dorsal-ventral defect in egg anatomy (Gonzalez-Reyes et al., 1997). Unrepaired DSBs in meiotic prophase I results in checkpoint activation that is Mei-41-dependent (Joyce and McKim, 2011). The checkpoint activation reduces Gurken protein levels, which influences dorsal-ventral patterning of the oocyte (Nilson and Schüpbach, 1999), however *smc5/6* mutants did not display a dorsal-ventral pattern defect suggesting that few persisting DSBs can evade the DNA damage checkpoint. This is in line with other data showing that a weak allele of *okr*, a DSB repair gene, is insufficient to activate the checkpoint despite a few unrepaired DSBs (Jang et al., 2003). Thus, we suggest that persisting meiotic breaks can be carried over and interfere with the rapid mitotic cleavage cycles causing premature failure of early embryogenesis that manifests as female subfertility.

**Fig. 9.**
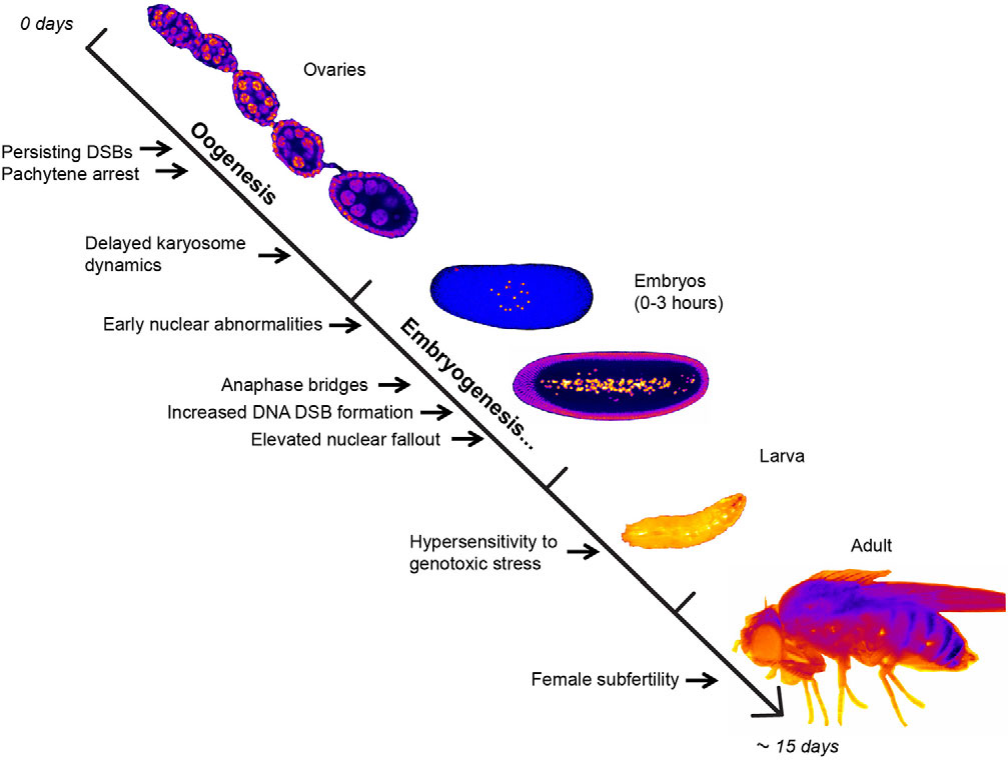
**Summary of Smc5/6 function in the developmental timeline.** The model represents a brief overview of *Drosophila* development emphasizing the developmental stages in which Smc5/6 performs an essential function. Smc5/6 absence during oogenesis results in persisting DSBs in the oocyte, pachytene arrest, and abnormal karyosome dynamics. Additionally, *smc5/6* mutants have various abnormalities in chromosome structure and behavior in conjunction with embryogenesis. The delay in embryogenesis is caused by increased anaphase-bridge formation that leads to elevated nuclear fallout. Absence of Smc6 in late developmental stages manifests as hypersensitivity to genotoxic stress and female subfertility, respectively. While *smc5/6* mutants acquire multiple chromosomal abnormalities during embryogenesis female subfertility appears to be primarily due to the meiotic defects occurring throughout oogenesis. Images are taken from our own experiments.

In summary, the data presented here describes a function of Smc5/6 during oogenesis and early embryogenesis of *Drosophila* (Fig. 9). A role of Smc6 in embryogenesis has been implicated earlier in mice, but no detailed description of function was provided since complete knockouts were lethal (Ju et al., 2013). Null mutants of *Drosophila* Smc5/6 were moderately viable and therefore enabled us to investigate their function at different developmental stages. Smc5/6 exhibited maternal function in chromosome maintenance during early embryogenesis. The reduced embryonic viability of *smc5/6* mutants was attributable to an impaired meiotic function of Smc5/6 during oocyte development, which was seen by increased X-NDJ, DSB persistence, pachytene arrest, and aberrant oocyte nucleus morphology. More investigations of Smc5/6 subunits are needed to address whether they share common phenotypes. Additionally, due to functional relations between the Smc5/6 and cohesin complex in yeast (Jeppsson et al., 2014), it will be interesting to further explore their mutual dependency during higher eukaryotic development. Defects in the cohesin complex and its partners are associated with cohesinopathies and were recently linked to cancer (Remeseiro et al., 2013). Hence, further studies of Smc proteins including Smc5/6 during *Drosophila* development could reveal SMC complex regulation and function and give insights into the complexity of human developmental disorders and tumorigenesis.

## MATERIALS AND METHODS

### *Drosophila* stocks and fly genetics

The *smc6* null allele was generated by imprecise P-element excision using the fly stock *SMC6*^P(EP)EP545^ (Bloomington, #BL17178) that carried a P-element insertion upstream of the 5´-UTR of *SMC6* on chromosome 3. The strategy and crossing scheme for isolation of excision mutants is reviewed in Hummel and Klämbt (Hummel and Klambt, 2008). PCR screening of 286 excisions was performed to detect chromosomal deletions in the *SMC6* protein-coding sequence. Genomic DNA from potential candidates were isolated and sequenced to define the deletions of the flanking genomic DNA, whereby one deletion allele was recovered and named *smc6*Δ^35^. The generated *smc6* null allele was backcrossed four times with wild-type (*w^1118^*) flies to remove any possible second site mutation(s).

To generate *SMC6*^6xHA^ transgenic flies the coding region of Smc6 including 409 bp upstream and 154 bp downstream sequences starting from the 5′-UTR and 3′-UTR, respectively, was synthesized (GeneScript USA Inc.) and cloned into the KpnI/EcoRI site of plasmid pattB. During the one-step synthesis of *SMC6* a 6xHA epitope tag was inserted in front of the stop codon. The generated construct was named pattB_SMC6 and integrated into the landing site pttP40 on the left arm of chromosome 2 (25C7) using the phiC31 integrase technology (Genetic Service Inc.).

To generate the rescue stock for the *smc6*Δ^35^ allele *SMC6*Δ^35^/*CyO; TM3*/*Vno* female virgins were crossed to *Sco/CyO*; *smc6*Δ^35^/*TM3* males. From that cross female virgins and male progeny with the genotype *SMC6*^6xHA^/*CyO*; *smc6*Δ^35^/*TM3* were collected and crossed to each other. From the resulting progeny with the genotype *SMC6*^6xHA^; *smc6*Δ^35^ a homozygous stock was generated.

For the time-lapse microscopy analysis homozygous *smc6*Δ^35^ female virgins were crossed to *HIS2AV-mRFP* males. From that cross *HIS2AV-mRFP*/ *smc6*Δ^35^ female virgins were collected and crossed to *Dr/TM3* males, from which potential *HIS2AV-mRFP smc6*Δ^35^/TM3 male progeny were collected. Single crosses were then performed crossing *HIS2AV-mRFP smc6*Δ^35^/TM3 males to *Dr/TM3* female virgins. After egg laying *HIS2AVmRFP smc6*Δ^35^/TM3 males were removed from the vials and the presence of the *smc6*Δ^35^ allele was analyzed by PCR (5′-cgcggcaatgtgctttatcatc-3′ and 5′-ggccctgcaacagtttgttctc-3′). If the presence of the *smc6*Δ^35^ allele was confirmed, vials were further incubated and female and male progeny with the genotype *HIS2AV-mRFP smc6*Δ^35^/TM3 were collected. The collected progeny were then crossed to each other and *HIS2AV-mRFP smc6*Δ^35^ female and male progeny were collected, from which a homozygous stock was generated.

Additional fly stocks used in this study were as follows: *w^1118^* (Bloomington, #BL3605), *C(1;Y)2,y^1^B^1^/0* and *C(1)RM, ras^1^/0* (Bloomington, #BL2510), *C[3]EN, st(1) cu(1) es/0* (Bloomington, #BL1117), *C(4)RM, ci(1) ey(R)/0* (Bloomington, #BL1785), *y^1^pn^1^cv^1^v^1^f^1^B^1^* (DGRC, #107867), w*; P[His2Av-mRFP]III.1 (Bloomington, #BL23650), *y/B(S)Y;mei-W68^2-0949^,cn bw/SM6* (W7, kindly provided by Kim S. McKim, Waksman Institute of Microbiology, Rutgers, The State University of New Jersey, Piscataway, NJ 08854, USA), and *w-; mnk^P6^* (kindly provided by William E. Theurkauf, Program in Molecular Medicine, University of Massachusetts Medical School, 373 Plantation Street, Worcester, Ma 01605, USA).

### Isolation of total RNA for RT-PCR (reverse transcriptase PCR)

Total RNA was extracted from 20 flies using Trizol (Invitrogen Life Technology) according to the manufacturer's instructions. The RNA was used directly for cDNA synthesis using the High-Capacity cDNA Reverse Transcription Kit (Applied Biosystems) followed by DNase I treatment (Invitrogen Life Technology). Real-time PCR using Fast SYBR Green PCR Master Mix (Applied Biosystems) was performed on a 7500 Fast Real-Time PCR System (Applied Biosystems). Primer used for RT-PCR: *SMC6* (5′-gatgaagtgaaccggaagtttattac-3′ and 5′-tagcctcgaccttcgtatcc-3′), *CG6204* (5′-ctttgaacgactcattgtggc-3′ and 5′-gatgtcctcgtattccttcactg-3′), and *RP49* (5′-gtcgccgcttcaagggacagta-3′ and 5′-gcgatctcgccgcagtaaac-3′).

### DNA damage sensitivity assay

For drug or ionizing radiation (IR) treatment first instar larvae were collected and placed into vials containing medium. Drug stocks were dissolved in DMSO and pre-added into the media to obtain a suitable working concentration [camptothecin (Sigma-Aldrich, final concentration 0.025 mM); hydroxyurea (Sigma-Aldrich, final concentration 8 mM)]. For IR, first instar larvae were irradiated at a dose of 40 Gray using a ^137^Cs radiation source (IBL 677, CIS Bio International). The survival index of a given genotype was calculated as the percentage by dividing the number of adult survivors by the number of adult survivors without drug or IR treatment.

### Embryo collection, fixation, and staining

Embryos were collected, heat fixated and stained as described by *Drosophila Protocols* (Sullivan et al., 2000). DNA was stained with PI (5 μg/ml). Embryos were subsequently dehydrated with the following 10-min ethanol washes regime: 50%, 70%, 80%, and two times in 100% ethanol. Solution was then replaced with methyl salicylate (Sigma-Aldrich) and stored at 4°C until mounting. For antibodies and dilutions see Table S4.

### Immunoblotting of embryo extract

Embryos were homogenized in 4× laemmli buffer (BioRad). Western blot performed as described by Nagarkar-Jaiswal et al. (2015). For antibodies and dilutions see Table S4. The membrane was developed and imaged according to manufacture instructions using Amersham ECL-kit (GE Healthcare).

### Confocal analysis

Zeiss Meta LSM510 laser confocal microscope was used to take 1 μm *z*-sections, which analyzed with ImageJ (NIH) software along with statistical analysis in Graphpad's Prism.

### Anaphase bridge quantification

Embryos were prepared as described under the section embryo collection, fixation, and staining. Embryos showing only late telophase nuclei (cycles 10-13) were examined. The presence of linkage between two DNA masses was scored as a bridging defect. Since DNA-bridges always connect two DNA masses the following equation was used to calculate anaphase-bridging frequency: [2×bridges/total number of nuclei]×100.

### Embryonic viability tests

Freshly hatched females and males were incubated at 25°C for 2-3 days. For egg collection flies were given a fresh apple juice agar plate to lay eggs for 30 min. The plate was discarded and replaced with a new agar plate. The flies were then allowed to lay eggs for 1 h before egg collection. Eggs were then counted and transferred into a vial. The vial was incubated at 25°C for 4 days, whereby the hatching rate was determined by counting the number of adult flies emerging from total amount of collected eggs.

### Temperature-shift experiment

Freshly hatched females along with males were accommodated to either 25°C or 18°C for 2-3 days before egg collection. The apple juice agar plates and vials were always temperature-adjusted beforehand to the desired developmental temperature. Flies laid eggs for 30 min on an agar plate, before replacing with collection plate, Flies laid eggs on collection plate for no more than 5 min, whereby eggs were immediately counted and transferred to a fresh vial. The process of egg laying and collection was performed within 10 min. The vial was incubated at either 25°C or 18°C. Hatching rate was determined as mentioned above.

### Nuclear mass measurement on fixed embryos

Two ImageJ macros defined the outer and inner rim. These macros were called zone 1 and zone 2, respectively. They had pre-set instructions that projected the *z*-stack into one image which identified the embryo as an object above background threshold intensity. The zones were masked one at a time, ImageJ then marked all identified nuclei in the relevant zone along with measurements of their integrated density, which were summed.

### Live imaging

Eggs no older than 10 min were collected from stocks of the genotypes *smc6*Δ^35^ P[His2Av-mRFP]III.1 and P[His2Av-mRFP]III.1 on apple juice agar plates. Embryos were dechorionated as described before and mounted with halocarbon oil 700 (Sigma-Aldrich, #H8898). The embryos were then immediately imaged for 3 h with a Zeiss LSM 510 Meta Laser Scanning Confocal Microscope. A total of eight *z-*sections at 5 μm interval were captured every 50 s using a 20× objective at resolution 1024×1024 pixels. Analysis was performed on ImageJ software (1.45s). *z*-stacks were projected and several projected images were combined into a time-lapse video.

### Oocyte extraction and staining

The ovaries were extracted and spread to individual ovarioles as described by Page and Hawley (2001) or Wong and Schedl (2006). Staining and washing procedure were performed described by Page and Hawley (2001). For antibodies and dilutions see Table S4. Ovaries were treated with PI (5 μg/ml) or DRAQ5 (5 μM) for 10 min. Dehydrated by a 10-min wash regime; 50%, 70%, 80%, and two times in 100% ethanol. Ovaries were stored and mounted at 4°C in methyl salicylate solution. γ-H2Av foci were counted stack-by-stack in germarial regions 2a, 2b and 3, such that only foci belonging to corolla-stained nuclei were counted. Oocyte determination by region 3 was also checked by corolla-stained nuclei.

### Karyosome area measurement

6 μm stacks were taken, which encompassed the entire karyosome of stage 3, or late stage 9/early stage 10 ovaries. A background threshold in ImageJ was applied. Using the wand-tracing function, ImageJ could identify and highlight the karyosome outline. Subsequently, the ImageJ measuring function acquired the area. *P*-values were calculated via GraphPad's Prism using Mann–Whitney two-tailed test.

### Meiotic crossover assay and autosomal NDJ test

To measure meiotic crossovers, males with the genotype *y pn cv v f; smc6*Δ^*35*^/+ were crossed to +/+; *smc6*Δ^*35*^/ *smc6*Δ^*35*^ females. The resulting female progeny *y pn cv v f*/ +; *smc6*Δ^*35*^/+ and *y pn cv v f*/ +; *smc6*Δ^*35*^/ *smc6*Δ^*35*^ were crossed to *y pn cv v f* males (DGRC, #107867) and the progeny were scored for all visible markers. The crossover rate was assayed in each of the four intervals and is represented by the number of map units (m.u.) per megabase (Mb). Map units were calculated by dividing the number of crossovers by the total number of progeny and multiplied by 100. Map units per megabase (m.u./Mb) were determined by dividing the map units by the definite physical distance between each visible marker within the intervals. Physical distances are taken from Flybase using the *Drosophila* genome build 6.05.

Meiotic autosomal NDJ was determined by crossing either wild-type (*w^*1118*^*) or *smc6*Δ^*35*^ females to *C(3)EN, st cu es/0* (Bloomington, #BL1117) and *C(4)RM, ci ey/0* males (Bloomington, #BL1785) for NDJ analysis of chromosome 3 and 4, respectively. From the cross that utilizes a compound 3-chromosome (*C[3]EN*) normal progeny (*3/C[3]EN*) did not survive, whereas only exceptional progeny due to nondisjunction such as nullo-3 or diplo-3 ova gave rise to *0/ C[3]EN* or *3^3/0* flies, respectively. The meiotic 3^rd^-NDJ frequency was calculated as the percentage from the exceptional progeny using the following equation: [Exceptional Progeny/ Total number of collected eggs]×100. From the cross that utilizes a compound 4-chromosome (*C[4]RM*) only nullo-4, but not diplo-4, ova were recovered. The frequency of meiotic 4^th^-NDJ was calculated by doubling this number and the following equation was used: [2×nullo-4/ total number of progeny]×100.

### X-NDJ assay

Meiotic X*-*NDJ was determined by crossing either wild-type (*w^1118^*), *SMC6*^6xHA^; *smc6*Δ^35^, *smc5*^P7E8^ or *smc6*Δ^35^ females to C(1;Y), *y*, ***B*** males (Bloomington, #BL2510). The males contained an attached X*^*Y chromosome that carries a dominant mutation (***B***) causing bar-shaped eyes. From the cross normal female progeny were ***B*** with red eyes, whereas normal male progeny were *B*^+^ with white eyes. Exceptional progeny such as diplo-X ova gave rise to XX/0 females whose eyes were *B*^+^ and white, whereas nullo*-*X ova gave rise to C(1;Y), *y*, ***B***/0 males whose eyes were ***B*** and red and had the additional patroclinous phenotype of yellow body. Diplo-X and nullo*-*X ova with the genotype C(1;Y), *y*, ***B***/XX and 0/0, respectively, did not survive. Meiotic X-NDJ was calculated as the percentage of progeny that arose from nondisjunction (*B*^+^ females and ***B*** males). The values for X-NDJ were corrected for loss of half of the diplo-X and half of the nullo-X ova according to the following equation: [2×Exceptional Progeny/(2×Exceptional Progeny+Normal Progeny)]×100.
